# Leader–follower UAVs formation control based on a deep Q-network collaborative framework

**DOI:** 10.1038/s41598-024-54531-w

**Published:** 2024-02-26

**Authors:** Zhijun Liu, Jie Li, Jian Shen, Xiaoguang Wang, Pengyun Chen

**Affiliations:** 1 Shenzhen MSU-BIT University, Shenzhen, 518172 China; 2 School of Mechatronical Engineering, Beijing Institute of Technology, Beijing, 100081 China; 3 School of Mechanical and Electrical Engineering, North University of China, Taiyuan, 030051 China; 4 Department of Advanced Technology, Norinco Group Aviation Ammunition Research Institute, Harbin, 150030 China; 5 School of Aerospace Engineering, North University of China, Taiyuan, 030051 China

**Keywords:** Engineering, Aerospace engineering, Mechanical engineering

## Abstract

This study examines a collaborative framework that utilizes an intelligent deep Q-network to regulate the formation of leader–follower Unmanned Aerial Vehicles (UAVs). The aim is to tackle the challenges posed by the highly dynamic and uncertain flight environment of UAVs. In the context of UAVs, we have developed a dynamic model that captures the collective state of the system. This model encompasses variables like as the relative positions, heading angle, rolling angle, and velocity of different nodes in the formation. In the subsequent section, we elucidate the operational procedure of UAVs in a collaborative manner, employing the conceptual framework of Markov Decision Process (MDP). Furthermore, we employ the Reinforcement Learning (RL) to facilitate this process. In light of this premise, a fundamental framework is presented for addressing the control problem of UAVs utilizing the DQN scheme. This framework encompasses a technique for action selection known as $\varepsilon$-imitation, as well as algorithmic specifics. Finally, the efficacy and portability of the DQN-based approach are substantiated by numerical simulation validation. The average reward curve demonstrates a satisfactory level of convergence, and kinematic link between the nodes inside the formation satisfies the essential requirements for the creation of a controller.

## Introduction

The development of unmanned aerial vehicles (UAVs) has been widely applied in various fields. UAVs can be combined with existing technologies to form more intelligent and efficient solutions that meet the needs of modern society. Aerial photography, unmanned warehousing, express logistics, rescue, and other fields have begun to explore the application of UAVs, and there will be more fields in the future that can unleash its potential^1^.

The integration of UAVs and artificial intelligence (AI) algorithms through the advancement of unmanned cluster technology has enabled the accomplishment of missions with enhanced efficiency and intelligence. Artificial intelligence algorithms can provide intelligent decision-making and control capabilities for UAVs^2,3^. For example, through deep learning technology, the autonomous perception and target recognition of UAVs can be realized, so that they can autonomously avoid obstacles and identify targets. Through the Reinforcement Learning (RL) algorithm, UAVs can be autonomously explored and learned in an unknown environment, achieving more flexible and intelligent task execution. Through the combination of UAVs and artificial intelligence algorithms, the collective intelligence and collaborative working ability of UAVs can be realized, and the task execution efficiency, ability to deal with complex environments, and autonomous decision-making capabilities of UAVs can be improved. This has important application value for some scenarios that require large-scale and complex task execution, such as disaster relief, agricultural plant protection, logistics distribution, etc.

To achieve the autonomous planning of UAVs in response to dynamic environmental conditions and facilitate collaborative efforts towards accomplishing mission objectives, Xu et al. implemented a novel MARL framework. The organization had adopted a strategic approach of centralized training coupled with decentralized execution, the utilization of the Actor-Critic network was employed to ascertain the execution activity and thereafter evaluated its efficacy. The new algorithm implemented three significant enhancements derived from the Multi-Agent Deep Deterministic Policy Gradient (MADDPG) method. Simulation results demonstrated a clear enhancement in learning efficiency, and there was an enhanced improvement in the operational safety factor in comparison to the preceding algorithm^4^. Hossein et al. proposed the implementation of a system that employs Deep Reinforcement Learning (DRL) as a means to tackle the difficulties associated with autonomous waypoint planning, trajectory tracking, and trajectory creation for multi-rotor UAVs. The present framework incorporated a DRL algorithm for the purpose of achieving optimal waypoint planning. The primary objectives of this algorithm were to minimize control energy expenditure and effectively navigated around obstacles within a specified area^5^. For the UAVs decision-making problem, Hu et al. introduced a solution for autonomous maneuver decision-making in air warfare involving two cooperating UAVs. Their approach utilized a Deep Q-Network (DQN) framework. The study conducted a simulation of the air warfare job using a dual-UAV olive formation. The findings indicated that the suggested technique demonstrated the potential to enhance the UAVs' ability to effectively defeat the enemy. Furthermore, it was observed that the proposed method outperformed the DQN method, specifically in terms of convergence speed, when priority sampling was not utilized^6^. Wang et al. conducted a comparative analysis of the merits and drawbacks associated with the utilization of DQN and Deep Deterministic Policy Gradient (DDPG) algorithms in the context of unmanned aerial vehicle (UAV) autonomous decision-making. The findings of the study indicated that the DDPG algorithm exhibited notable benefits in terms of action continuity and reduced decision time. However, it was shown that the DQN had superior decision-making capabilities when compared to other models of similar complexity^7^. Yang et al. presented a novel approach to address the challenges associated with the UAV transformation problem. Their method involved utilizing an enhanced DQN algorithm to handle the intricate system structure and extensive computational requirements typically encountered in conventional multiple UAV formation transformation techniques. Simulation results demonstrated that the proposed strategy effectively improved the efficiency of transforming multi-UAV formations. Furthermore, the approach exhibited favorable characteristics of generalization and practicability^8^. For the UAV landing maneuver problem, Rodriguez-Ramos et al. introduced a novel DDPG algorithm as a solution to the problem of executing UAV landing operations on a mobile platform. Numerous simulations had been conducted under diverse settings, encompassing simulated and actual flights, thereby establishing the broad applicability of the methodology^9^. For the UAV pursuit-evasion problem, Moulay et al. employed DRL techniques to forecast the appropriate actions for the follower UAV in order to effectively monitor the movements of the target UAV. The efficacy of the algorithms under consideration was proved in outdoor tracking scenarios by the utilization of actual UAVs^10^. Singh et al. extended the Actor-Critic model-free MADDPG algorithm, which was designed for continuous areas, in order to tackle the pursuit-evasion problem. The algorithm was successfully applied, and the subsequent analysis of the results demonstrated that the pursuers were able to acquire a viable control plan for effectively capturing the evaders^11^.

During the execution of multiple tasks, UAVs collaborative control refers to the collaborative work to achieve efficient completion of tasks. In the context of collaborative control of UAVs, it is essential to acknowledge that each UAV is associated with non-linearity. Consequently, the attainment of attitude control for UAVs necessitates the utilization of nonlinear control methodologies^12^. Nevertheless, when it comes to real-world scenarios, the modeling techniques fail to provide a true depiction of the attributes exhibited by UAVs. The precise aircraft system model typically exhibits time-varying behavior, complexity, and non-linearity. Random factors, like errors in sensors and disturbances in the environment, can provide challenges when attempting to create accurate models. The utilization of conventional control approaches is significantly constrained by this factor. The utilization of the model-free RL approach as a potential solution to the aforementioned paradox has garnered growing interest^13^. The utilization of DRL-based collaborative control has experienced a notable rise in several domains involving multiple agents, including multi-robot systems, UAVs, satellite formations, unmanned surface vessels (USVs), and other related fields. The utilization of control design methodology incorporating DRL technology enables the achievement of collaborative control among UAVs without necessitating a precise system model.

The overall approach suggested by Zhou et al. aimed to integrate DRL with a simulation environment for UAVs. The entire system included of the DRL algorithm utilized for attitude control, the Robot Operation System (ROS) packing technique employed to establish a connection between DRL and the PX4 controller, and a Gazebo simulator that replicated the real-world environment. The efficacy of the proposed framework was substantiated by the experimental findings^14^. Zhao et al. introduced a computational guidance approach based on DRL to address the issue of preventing collisions among a formation of fixed-wing UAVs operating within a confined airspace. The findings of simulated experiments conducted on several cases demonstrate that the utilization of a real-time guiding method can significantly decrease the likelihood of collisions among UAVs during flight, even when dealing with a substantial number of aircraft^15^. Moon et al. introduced a new DRL method to effectively manage the movement of several UAVs in order to monitor multiple First Responders (FRs) in complex three-dimensional (3D) environments that contain barriers and occlusions. The simulation findings indicated that the UAV controller based on DRL offered a precise target-tracking solution with minimal computational overhead^16^. Liu et al. proposed the utilization of new DRL techniques for UAV control. The authors presented an innovative and very efficient approach rooted in DRL. The simulation results had proven that the method based on DRL consistently and considerably beat two generally employed baseline methods in terms of fairness, coverage, and energy usage^17^. Wang et al. proposed a framework for mobile edge computing (MEC). The suggested framework involved many UAVs with distinct trajectories flying over a designated area to provide support to user equipment located on the ground. The trajectory control algorithm presented by the authors was grounded on the MADDPG technique, which aimed to autonomously manage the trajectory of each UAV. The simulation results demonstrated that the suggested approach exhibited significant performance advantages compared to conventional algorithms^18^. Zhang et al. introduced an enhanced DDPG method to address the UAVs control problem in route following scenarios. The efficiency of the proposed strategy was demonstrated by the execution of simulation experiments^19^. Wang et al. examined a control approach based on RL for USVs. This technique aimed to develop a motion control policy that could effectively counteract wave disturbances. The findings of the simulation experiment indicated that the control strategy, after being taught, shown a satisfactory ability to manage the unmanned surface vehicle (USV) in front of wave disturbances^20^. Wan et al. presented a new DRL approach together with a robust DDPG algorithm. Their research was to design a controller capable of enabling an UAV to navigate reliably in dynamic and uncertain situations. The learning-based controller was implemented using an actor-critic architecture, enabling it to effectively execute a dual-channel continuous control of the UAV, specifically controlling the roll and speed. The training experiments demonstrated significant enhancements in terms of convergence speed, convergence effect, and stability^21^. Xu et al. advocated the utilization of the DDPG algorithm for the purpose of achieving autonomous morphing control and the adoption of the MADDPG algorithm to facilitate cluster cooperative operations, so enabled the attainment of autonomous and cooperative fighting capabilities in morphing UAV clusters. The objective was to achieve intelligent cluster combat and gain control over the future air combat initiative^22^. Tožička et al. devised a control system for a fleet of several UAVs by leveraging insights from the latest advancements in the field of deep reinforcement learning. The control policy for this study was selected to be a deep Convolutional Neural Network (CNN) with a linear output layer. This choice was made based on the extensive range of applications that CNN had demonstrated. The training of the control policy was conducted in a simulated environment including five UAVs. The control policy was implemented and executed on a fleet consisting of five DJI Mavic Pro drones, yielding satisfactory results^23^.

UAVs, discussed in this paper utilizing a leader–follower framework, wherein a single UAV assumes the role of the leader while other UAVs act as followers, collectively forming a formation. Given the control design requirements that have emerged, it is imperative for the follower to consistently uphold the leader's relative distance and adapt to any associated variations in parameters, even in the event of leader maneuvering. When examining the modeling characteristics of collaborative control problems in leader–follower UAVs, it becomes evident that a DRL-based algorithm is the most suitable option for addressing the challenges posed by the UAVs dynamic nature and the uncertainties present in their flight environment. The DQN-based approach, which is widely employed in the field of DRL, has the capability to acquire intricate decision-making methods without any prior knowledge. Moreover, it has the capability to efficiently enhance performance in large state spaces. This study focuses on the development of a cooperative control algorithm for UAVs based on the features of their flight environment. In Section Method, we firstly provides an illustration of the environment in which UAVs operate. The dynamic model is a representation of the joint state of an intelligent UAV system. This model encompasses the velocity, relative locations, heading angles, and rolling angles of both the leader and the followers. Furthermore, the collaborative control process of leader–follower UAVs is characterized as a Markov decision process (MDP) model. This will go into the action space, state space, and reward function associated with this model. Then, we will describe the fundamental structure of the control problem for leader–follower UAVs, utilizing the DQN approach. The proposed approach includes the strategy of $\varepsilon$-imitation for action selection, the creation of Q-network, and the specification of algorithm details. In this study, the suggested DQN-based method is subjected to numerical simulation tests in order to validate its convergence and portability. The main contribution of this study is illustrating a collaborative framework that utilizes an intelligent deep Q-network to regulate the formation of leader–follower UAVs. This framework addresses the challenges posed by the highly dynamic and uncertain flight environment of UAVs. The study proposes a novel intelligent control strategy for the cooperative control of UAVs, utilizing a DQN algorithm. The proposed system joint states encompass the relative position, heading angle, and rolling angle in the formation of UAVs, as defined in the environmental context. Additionally, the study modifies a MDP model to depict the collaborative control process of UAVs, using the fundamental scheme of RL. With the analysis of the simulation results, it showed a reasonable convergence in the UAVs collaborative control process. The work mentioned in this manuscript will provide a new view for the UAVs collaborative control problem, and the control strategy training in the numerical simulation environment can be directly transferred to the hardware in the loop simulation system without too much parameter adjustment.

## UAVs dynamics and MDP framework

### Motion equation of the UAV

Given the assumption that the UAV maintains a constant altitude, the mathematical representation of the system can be reduced to four independent variables, hence simplifying the model to four degrees of freedom. In order to make up for the loss caused by simplification and consider the influence of environmental disturbance, randomness is introduced into each sub-state such as roll and airspeed, and the obtained stochastic UAV kinematics model is shown as Eq. (1).

1 $$\dot{\xi } = \frac{d}{dt}\left\{ {\begin{array}{*{20}c} x \\ y \\ \psi \\ \phi \\ \end{array} } \right\} = \left\{ {\begin{array}{*{20}c} {V\cos \psi + \eta_{x} } \\ {V\sin \psi + \eta_{y} } \\ { - \left( {a_{g} /V} \right)\tan \phi + \eta_{\psi } } \\ {f\left( {\phi ,\phi_{d} } \right)} \\ \end{array} } \right.$$

where $\left( {x,y} \right)$, $\psi$, $\phi$ and $V$ are the position, the heading angle, the rolling angle, and the velocity of the UAV, respectively. $a_{g}$ is the gravity acceleration. $\eta_{x}$, $\eta_{y}$ and $\eta_{\psi }$ represent the disturbances of the state variable. All data points in the sample adhere to the normal distribution. The statistical measures of central tendency and dispersion, specifically the mean values and variance, are provided in Table 1. $f\left( {\phi ,\phi_{d} } \right)$ represents the relationship between the rolling angle $\phi$ and its desired value $\phi_{d}$. Second-order system response is used to simulate the dynamic response of UAV rolling channels.

**Table 1:** Arguments of the DQN method.

Argument	Value	Argument	Value
Number of followers	2	Return discount factor $\gamma$	0.95
Maximum action candidates $\phi_{\max }$ (°)	15	Update period of the target network $K$	10^3^ → 10^4^
Threshold of heading angle $\psi_{g}$ ( )	20	The capacity of experience replay pool $N$	10^5^
Threshold of the rolling angle $\phi_{bd}$ ( )	30	Mini-batch size $n_{e}$	32
Inner radius $d_{I}$ (m)	40	Total episodes $N_{s}$	5 × 10^4^
Outer radius $d_{O}$ (m)	60	Each episode time $t_{E}$ (s)	60
Time step $\Delta t$ (s)	1.0	Episodes number $N_{Avg}$ to calculate average total reward	100
Exploration probability $\varepsilon$	1 → 0.1	$\eta_{\psi }$’s mean value and variance $\left( {\overline{\eta }_{\psi } ,\sigma_{\psi } } \right)$	(0.0,1.0)
Learning rate $\lambda$	0.01	$\eta_{x}$’s mean value and variance $\left( {\overline{\eta }_{x} ,\sigma_{x} } \right)$	(0.0,1.0)
Adjust factor $\omega$	0.05	$\eta_{y}$’s mean value and variance $\left( {\overline{\eta }_{y} ,\sigma_{y} } \right)$	(0.0,1.0)

### UAVs system model

The utilization of the leader–follower control method is a significant technical strategy that plays a crucial role in ensuring the coherence and effectiveness of collaborative control systems^24,25^. The leader is an exceptional individual. As the individual assuming leadership within the formation, they possess the authority to determine and direct the trajectory of the formation, so that it is not affected by external influences. Nevertheless, the follower is not obligated to perceive the target data of the formation, but rather solely relies on the information provided by the leader^26^. Hence, a drawback of the leader–follower control approach relates to the inherent independence between the leader and the follower, making it challenging to obtain feedback on tracking errors from the follower.

**Figure 1:**
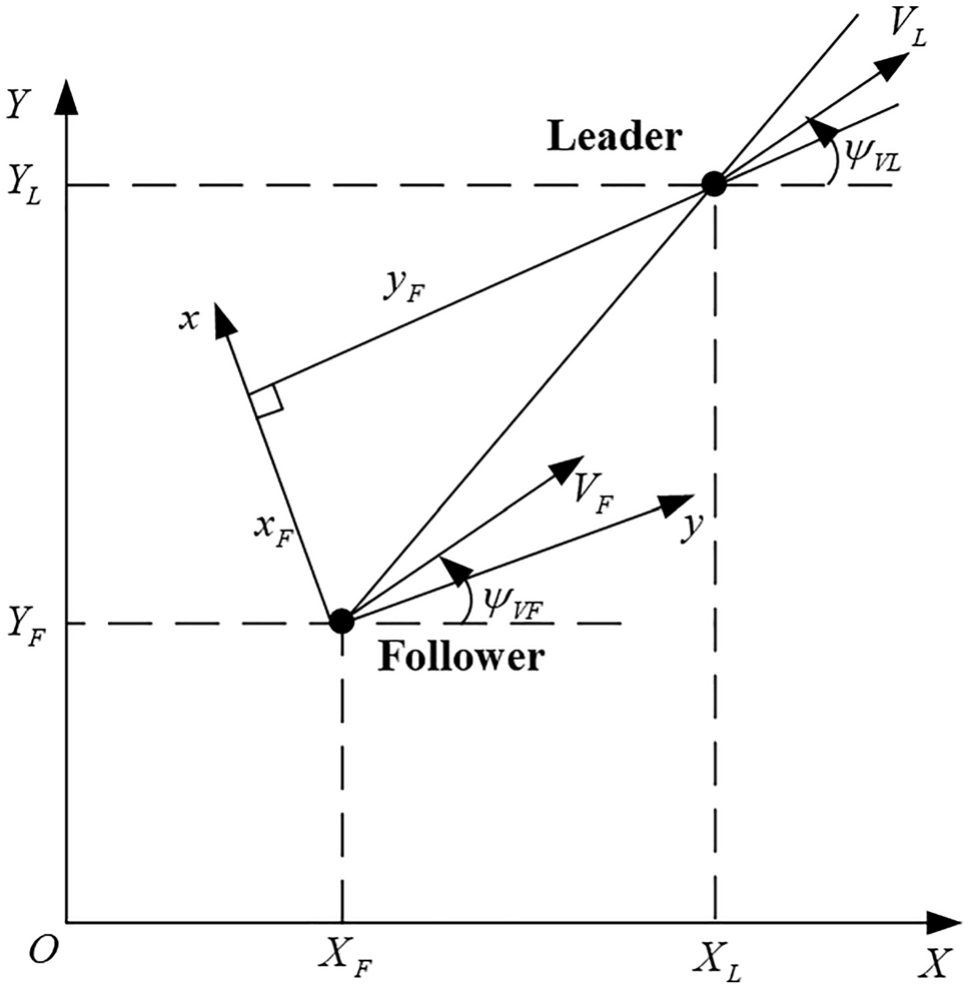
The relationship between the leader and the follower within the inertial coordinate system.

In order to depict the relative positioning of the leader–follower arrangement of UAVs, a coordinate system is developed with the follower UAV serving as the reference point, as seen in Fig. 1^27^.

In Fig. 1, the coordinates $X_{L}$ and $Y_{L}$ represent the inertial system of the leader. $X_{F}$ and $Y_{F}$ represent the inertial system of the follower. In the follower’s velocity coordinate system, $x_{F}$ and $y_{F}$ represent the relative distances between the leader and the follower. $V_{L}$ and $V_{F}$ represent the velocity of the leader and the follower. $\psi_{VL}$ and $\psi_{VF}$ denote the heading angles of the leader and the follower, respectively.

The control of the UAV is achieved by changing the roll angle setting value. The control strategy periodically updates the roll command at a frequency of one time per second, while the self-driving instrument effectively executes the low-level closed-loop control within the specified interval^28^. With consideration of the data presented in Fig. 1, the states $S$ of UAVs, which depict the relative link between the leader and the follower, can be represented as^29^:

2 $$\left\{ \begin{gathered} S_{1} = \psi_{VF} - \psi_{VL} \hfill \\ S_{2} = \phi_{L} \hfill \\ S_{3} = \phi_{F} \hfill \\ S_{4} = \phi_{d}^{l} \hfill \\ \left[ {\begin{array}{*{20}c} {S_{5} } \\ {S_{6} } \\ \end{array} } \right] = \left[ {\begin{array}{*{20}c} {x_{F} } \\ {y_{F} } \\ \end{array} } \right] = \left[ {\begin{array}{*{20}c} {\cos \psi_{VL} } & {\sin \psi_{VL} } \\ { - \sin \psi_{VL} } & {\cos \psi_{VL} } \\ \end{array} } \right]\left[ {\begin{array}{*{20}c} {X_{F} - X_{L} } \\ {Y_{F} - Y_{L} } \\ \end{array} } \right] \hfill \\ \end{gathered} \right.$$

where $S_{1}$ are the differences of the heading angles between the leader and the follower. $S_{2}$ and $S_{4}$ are the leader’s rolling angle and its desired value, $S_{2}$ represents the follower’s rolling angle. $S_{5}$ and $S_{6}$ are the differences of the relative position in $x,y$ direction between the leader and the follower. During actual flying operations, the control commands issued by the leader will be modified in response to the prevailing conditions on the battlefield. To enhance the adaptability of the model to dynamic input uncertainty, control instructions will be either constant or randomly produced through user functions in Results Section.

### MDP model for UAVs collaborative control

**Figure 2:**
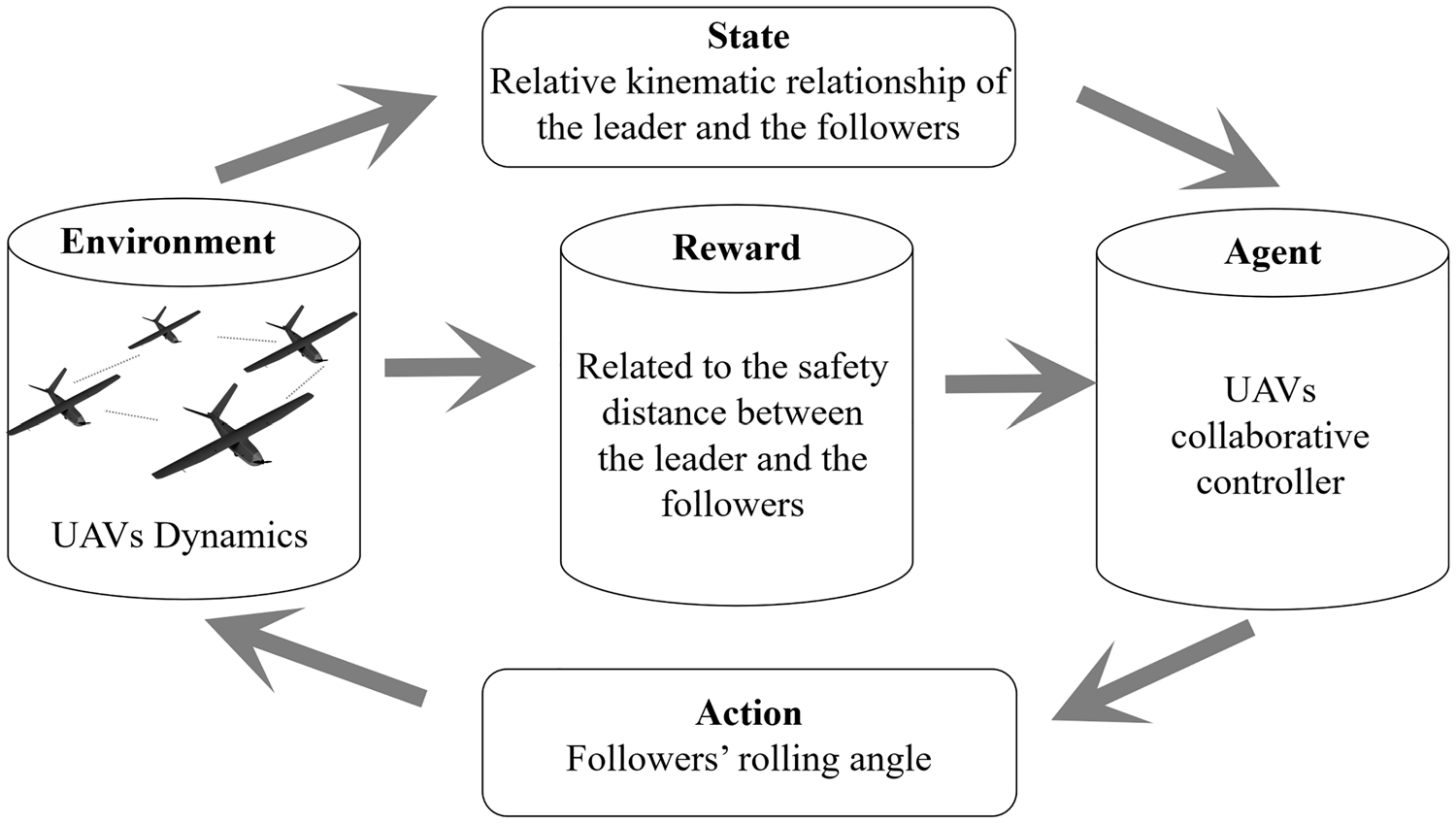
The framework of collaborative control for UAVs based on MDP.

Based on the aforementioned material, it is evident that the control issue pertaining to UAVs can be characterized as a multi-step decision-making problem. At its essence, this problem entails the selection of the suitable control command for the roll angle, as well as the determination of the optimal timing for executing and releasing the order decisions. This work presents a novel approach for addressing the control issue in collaborative control of UAVs, utilizing an intelligent and efficient control mechanism. The activities of UAVs have been reinterpreted within the context of MDP. The fundamental MDP paradigm is depicted in Fig. 2.

The representation of a discrete MDP can be achieved through the utilization of a quintile array denoted as $\left\{ {S,A,R,P,J} \right\}$. The state space, denoted as $S$, is partitioned based on the attitude and relative position of the leader and followers. The action space $A$ consists of the control instructions for the follower's rolling angle. $R$ represents the return values associated with actions and states. $R$ illustrates the transition percentage between states, and $J$ represents the optimization objective function of the control decision. The properties of a discrete MDP are as follows.

3 $$\begin{gathered} p\left( {s_{t + 1} = s_{j} \left| {s_{t} = s_{i} } \right.,a_{t} = a_{k} ,s_{t - 1} ,a_{t - 1} ,...,s_{0} ,a_{0} } \right) \hfill \\ = p\left( {s_{t + 1} = s_{j} \left| {s_{t} = s_{i} } \right.,a_{t} = a_{k} } \right) \hfill \\ = p_{ij} \left( {a_{k} } \right) \hfill \\ \forall s_{i} ,s_{j} \in S,a_{k} \in A,\forall t \ge 0 \hfill \\ \end{gathered}$$

where $p_{ij} \left( {a_{k} } \right)$ represents the probability of transitioning from state $s_{i}$ to state $s_{j}$ when the action $a_{k}$ is executed in the given state.

The learning effect of the formation controller in the discrete MDP model is directly influenced by the range and precision of the discrete parameters in the state space $S$. The selection of state space parameters for the formation MDP model in the warfare process of UAVs encompasses aspects such as the relative position and attitude between the leader and the follower. The other four parameters $A,R,P,J$ of the MDP model are primarily developed based on the intended mission target. Action space $A$ incorporates the rolling angle's operation. The reward function $R$ is built by utilizing UAVs to measure the distance values between the real-time positions of various members inside a formation. The transition probability $P$ is contingent upon the precise location of the UAV subsequent to the execution of the action. The objective function $J$ represents the total return value. As the action selection strategy, $J^{*}$ represents the optimal return value:

4 $$J* = \mathop {\max }\limits_{\pi } J = \mathop {\max }\limits_{\pi } E\left( {\sum\limits_{t = 0}^{\infty } {\gamma^{t} r_{t} } } \right)$$

where $\gamma \in \left( {0,1} \right)$ illustrates the return discount factor, $r_{t}$ indicates the return value of time $t$.

#### State space

The representation of UAVs can be achieved through the utilization of a multidimensional array. The establishment of a collaborative control problem inside the leader–follower topology necessitates careful consideration of the relative link between the leader and the follower. Factors such as heading difference and distance play a pivotal role in shaping the formulation of the control strategy. System state is used to represent the state space, which serves to describe the pose relationship and relative spatial location between the leader and the follower. In real engineering applications, based on the relative position relationship, the determination of the control command of the leader is contingent upon the flight control system. The primary focus of this study pertains to the development of a collaborative control architecture. In order to enhance the model's ability to handle diverse inputs, a random function is employed to create the control instruction of the leader during the training process of the DQN. This approach aims to imitate the inherent uncertainty associated with system input. The state space of a MDP scheme for UAVs can be denoted as $S = \left\{ {S_{1} ,S_{2} ,S_{3} ,S_{4} ,S_{5} ,S_{6} } \right\}$, as stated in Eq. (2).

#### Action space

The manipulation of the UAV is achieved through the alteration of its rolling angle. The control approach involves updating the control command at a frequency of one time per second, and the lower closed-loop control is executed by the autonomous system within this specified duration. The action space encompasses the rolling angle command of the follower UAV, taking into account the UAV's maximum acceleration and the need to prevent abrupt changes in control commands that could disrupt the flight of the UAV. On one side, it is advantageous for the followers to closely align with the current status of the leader's movement. Conversely, it is imperative to mitigate the inherent instability of the UAVs structure.

The set of possible actions, denoted as $A$, that can be taken by the followers can be represented as:

5 $$A = \left[ {\begin{array}{*{20}c} { - \phi_{\max } ,} & {0,} & { + \phi_{\max } } \\ \end{array} } \right]$$

where $\phi_{\max }$ denotes the upper limit of potential actions for the rolling angle of the followers.

The expected action for the subsequent time step is depicted as:

6 $$\phi_{d} = \left\{ {\begin{array}{*{20}c} {\begin{array}{*{20}c} {\phi_{bd} } & {\phi + a_{\phi } } \\ \end{array} > \phi_{bd} } \\ {\begin{array}{*{20}c} { - \phi_{bd} } & {\phi + a_{\phi } } \\ \end{array} < - \phi_{bd} } \\ {\begin{array}{*{20}c} {\phi + a_{\phi } } & {otherwise} \\ \end{array} } \\ \end{array} } \right.$$

where $a_{\phi }$ has been selected based on the control demand of the followers. $\phi_{bd}$ represents the thresholds associated with the follower's rolling angle.

#### Reward function

**Figure 3:**
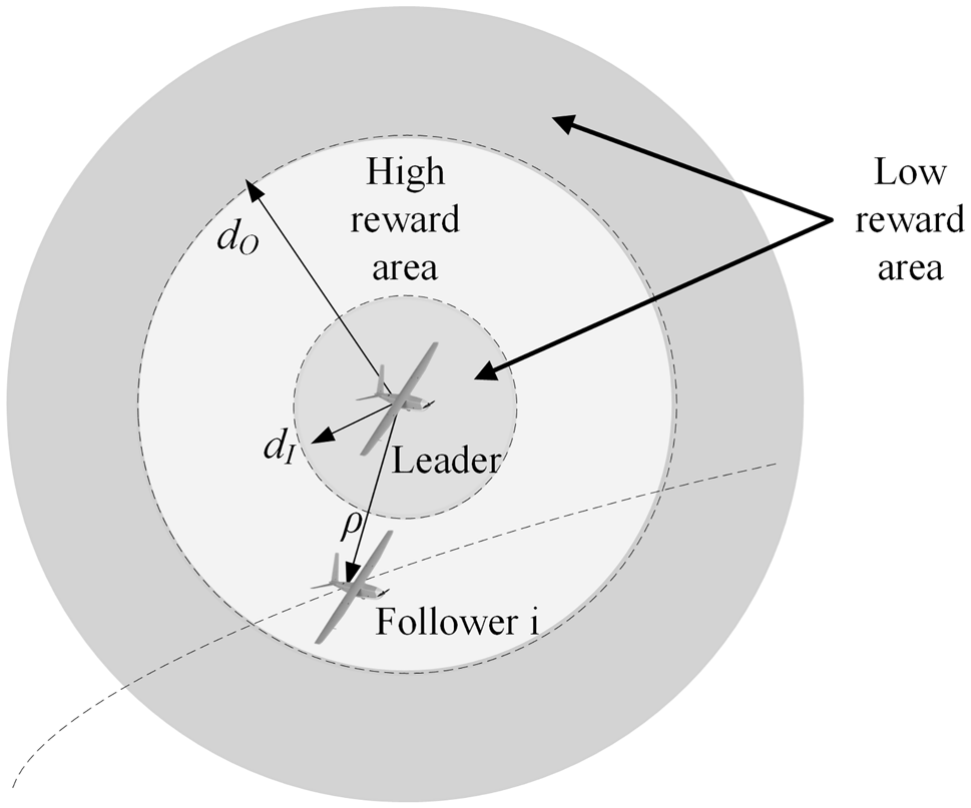
The scheme for collision avoidance in formations of UAVs.

In order to ensure proper configuration maintenance, it is imperative that each node inside the formation maintains a safe distance from its neighboring nodes. Insufficient space between nodes may result in collisions occurring among these neighboring entities. In the event that the distance is considerable, the delay time in communication will give rise to additional malfunctions^30^. Figure 3 illustrates a collision avoidance and reward evaluation scheme based on the intended elevated reward and the range $\left( {d_{O} ,d_{I} } \right)$ between the leader and the following UAVs. Each node will receive a reward value from the leader based on the proximity to its neighboring nodes. The individuals inside the group will modify their conditions in accordance with the reward values provided.

The construction of an acceptable reward function is crucial in the field of reinforcement learning. The cost function for collaborative control of UAVs is formulated and the reward function has been defined^31^. The reward function primarily takes into account the distance of UAVs, as depicted in Fig. 3. The value of reward limits ensure that the followers remain within the distance of the UAVs once the action has been executed. The reward function is depicted as:

7 $$\left\{ \begin{gathered} r = - {\text{Cos}} t \hfill \\ {\text{Cos}} t = \max \left\{ {D,\frac{{d_{I} \left| {s_{1} } \right|}}{{\pi \left( {1 + \omega D} \right)}}} \right\} \hfill \\ D = \max \left\{ {d_{I} - \rho ,0,\rho - d_{O} } \right\} \hfill \\ \rho = \sqrt {s_{5}^{2} + s_{6}^{2} } \hfill \\ \end{gathered} \right.$$

where $r$ represents the immediate reward. The inner radius and outer radius in Fig. 3 are denoted as $d_{I}$ and $d_{O}$, respectively. $D$ illustrates the spatial separation between the follower and the circular object. The adjust factor, denoted as $\omega$, is employed to modify the weight of $D$. $\rho$ illustrates the spatial separation between the leader and the follower.

### DQN control method

#### Fundamental framework

In the context of UAVs, it is observed that subordinate units obtain the relevant system status information from the commanding unit. The selection of actions in the control system is determined by the action-selecting strategy. The value of reward function is then calculated based on the feedback received from the updated system state information, which is obtained after the execution of the selected action. This study aims to reassess the benefits and drawbacks of the action plan by using the real-time rewards obtained by UAVs and optimizing the cumulative return. The Q-learning algorithm, within the context of this theoretical framework, is responsible for storing and estimating the action value function of the follower in various states within the MDP model. Additionally, it utilizes the real-time system state information provided by the pilot to iteratively renew the action value function. This iterative process aims to solve the optimal sequential decision-making problem associated with the follower actuator.

The value function estimation $Q\left( {s_{t} ,a_{t} } \right)$ of the action $a_{t}$ executed by the follower in state $s_{t}$ is determined:

8 $$Q\left( {s_{t} ,a_{t} } \right) = E\left( {\sum\limits_{t = 0}^{\infty } {\gamma^{t} r_{t} \left| {s_{0} = s_{t} ,a_{0} = a_{t} } \right.} } \right)$$

where $s_{0}$ represents the initial state of UAVs, $a_{0}$ illustrates the first action of the follower.

Based on the pertinent theory in the field of operations, $Q\left( {s_{t} ,a_{t} } \right)$ can be observed to satisfy the Bellman equation as follows:

9 $$Q\left( {s_{t} ,a_{t} } \right) = \sum\limits_{{s_{t + 1} }} {\left[ {p\left( {s_{t} ,a_{t} ,s_{t + 1} } \right)r\left( {s_{t} ,a_{t} ,s_{t + 1} } \right)} \right]} + \gamma \sum\limits_{{s_{t + 1} ,a_{t + 1} }} {\left[ {p\left( {s_{t} ,a_{t} ,s_{t + 1} } \right)Q\left( {s_{t + 1} ,a_{t + 1} } \right)} \right]}$$

where $p\left( {s_{t} ,a_{t} ,s_{t + 1} } \right)$ shows the probability of state $s_{t}$ transition to state $s_{t + 1}$ with actions $a_{t}$. $r\left( {s_{t} ,a_{t} ,s_{t + 1} } \right)$ represents the return value of state $s_{t}$ transition to state $s_{t + 1}$ with actions $a_{t}$.

The optimal strategy $Q\left( {s_{t} ,a_{t} } \right)$ of Q-learning relates to maximize the accumulative return value, hence the strategy may be formulated as:

10 $$\pi *\left( {s_{t} } \right) = \arg \begin{array}{*{20}c} {} \\ \end{array} \mathop {\max }\limits_{{a_{t} }} Q\left( {s_{t} ,a_{t} } \right)$$

In the field of RL, agents engage in ongoing interactions with their environment through a process of trial and error. The objective of this iterative process is to acquire an optimal strategy that maximizes the cumulative reward obtained from the environment^32^. In the Q-learning method, the determination of the Q-value function enables the identification of an optimal strategy. This is achieved by employing the greedy approach, where the agent selects the action indicated by the maximum Q-value at each time step. The Q-learning technique is commonly employed and very straightforward to implement. Nonetheless, it is still confronted with the challenge of the dimensional disaster. The approach commonly use tabular representation for storing Q values, rendering it unsuitable for reinforcement learning issues characterized by high-dimensional or continuous state spaces^33^.

The utilization of deep neural network (DNN) as function approximators for estimating Q values has emerged as a viable approach for addressing the aforementioned challenge^34^. Minh et al. demonstrated the utilization of CNN and empirical playback technology for the implementation of a Q-learning algorithm, exemplifying the application of a DQN method^35^. To mitigate the uncertainty of the neural network approximation function, a distinct target network was employed to generate Q values. This approach aimed to minimize the correlation between the predicted Q value, which is the output of the main network, and the target Q value, which is the output of the target network.

Based on the equation presented as Eq. (8), it is necessary to construct the optimal policy subsequent to the attainment of the maximum Q-function. By employing the recursive framework, the Q-function can be iteratively renewed as^36^,

11 $$Q\left( {s_{t} ,a} \right) = Q\left( {s_{t} ,a} \right) + \lambda \left[ {r_{t + 1} + \gamma \mathop {\max }\limits_{a} Q\left( {s_{t + 1} ,a} \right) - Q\left( {s_{t} ,a} \right)} \right]$$

where $\lambda$ represents the learning rate.

The target Q value is illustrated as:

12 $$y_{t}^{DQN} = r_{t + 1} + \gamma \mathop {\max }\limits_{a} Q\left( {s_{t + 1} ,a,\theta^{ - } } \right)$$

where $\theta^{ - }$ shows the parameter of the target network.

The minimize loss function can be shown as:

13 $$L\left( \theta \right) = {\rm E}\left[ {\left( {y_{t}^{DQN} - Q\left( {s_{t + 1} ,a_{t} \left| \theta \right.} \right)} \right)^{2} } \right]$$

As demonstrated by the DQN, the disparity between the assessed Q value of the main network and the Q value output of the target network is utilized to dynamically alter the parameters of the main network. In contrast to the real-time renewed parameters of the main network, the parameters of the target network are renewed at regular intervals of $K$ time steps. The target network parameters are updated by copying the main network parameters at regular intervals of $K$ time steps.

**Figure 4:**
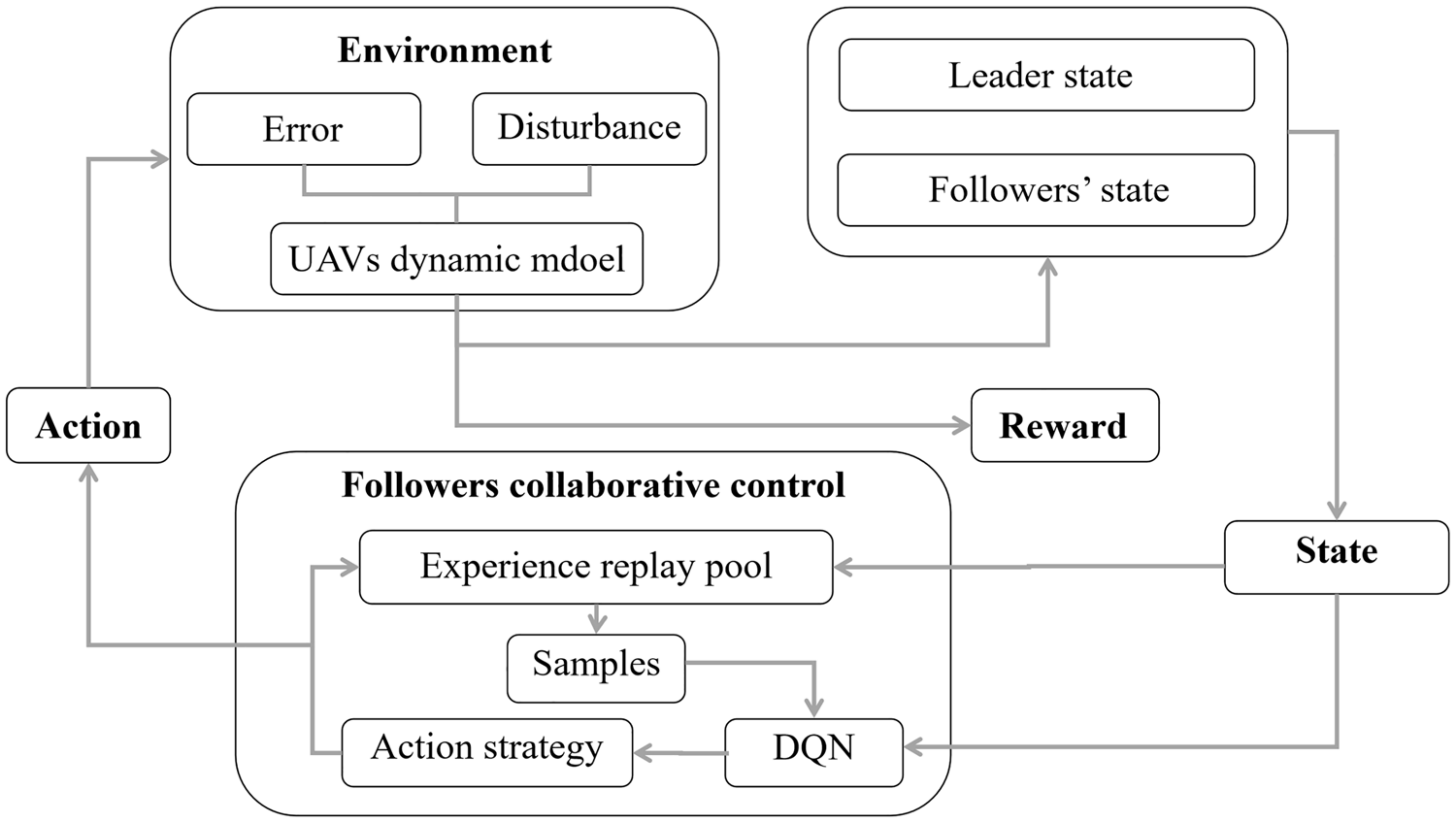
The framework for collaborative control of UAVs based on the DQN control algorithm.

In this part, we propose a control strategy for collaborative control of UAVs based on the DQN algorithm. The DQN algorithm is an innovative adaptation of Q-learning that integrates reinforcement learning with artificial neural networks^35^. In order to mitigate the instabilities that arise from approximating the action value function (Q function) using neural networks, the DQN approach incorporates the utilization of a periodically renewed separate target Q-network and an experience replay mechanism. DQN algorithm has demonstrated successful applications in several sectors, including agriculture, communication, healthcare, and aerospace engineering^37^. The structure of the control algorithm based on DQN is depicted in Fig. 4.

As depicted in Fig. 4, the followers are assigned to the agents within the framework of RL. The agents acquire the control method and modify the network arguments through ongoing interactivity with the environment. The followers receive both the state message of the leader and their state information. The state message is combined to generate a joint system state $S$, which is then fed as input of the DQN process. The action selection policy, referred to $\varepsilon$-imitation where $\varepsilon$ indicates the exploration ratio, determines the follower's rolling angle based on DQN’s output. The action instructions issued by the leader and the followers are utilized as inputs in the kinematics model of UAVs to determine the state of both the leader and the followers at the subsequent time step. The value of the reward function, denoted as $R$, as well as the system state, denoted as $S{\prime}$, at the next time step can also be obtained. $\left( {S,A,R,S^{\prime}} \right)$ values throughout the interaction process are preserved in the experience pool. During each iteration, the experience pool is subjected to random sampling, and afterwards, the network arguments of the DQN are modified. Once the predetermined number of time steps is reached in each round, the ongoing episode concludes and the subsequent episode commences.

#### Action strategy

In order to enhance the learning productivity of DQN during the training phase, a novel action favour approach called $\varepsilon$-imitation is proposed. This method combines the $\varepsilon$-greedy strategy and the imitation strategy, aiming to strike a balance between exploration and exploitation in the learning process^31^. The imitation method involves the follower selecting its control instruction based on the control demands related to relative distance. The essential concept of this method is that when followers make a selection from the action space with a chance of 1 − $\varepsilon$, the chosen action is determined by the expected relative distance between the leader and the follower, as outlined in Eq. (2). When the separation between the leader and the follower exceeds the designated safety limits $\left( {d_{O} ,d_{I} } \right)$, a derivative action $A_{\max }$ is selected from the available set of actions in order to mitigate the danger of collision. In the event that the relative distance is deemed to be within a secure range, it is advisable for the follower to sustain their present conditions, resulting in a state of inaction or zero action. The utilization of $\varepsilon$-imitation action selection strategy in the context of topology maintenance in UAVs flying has several advantages, including the reduction of follower blindness during the first exploration period. Additionally, this approach mitigates the occurrence of invalid explorations, enhances the quantity of positive samples within the experience pool, and contributes to the optimization of training efficiency.

$\varepsilon$-imitation action selection strategy can be observed in Algorithm 1^37^.

**Figure:**
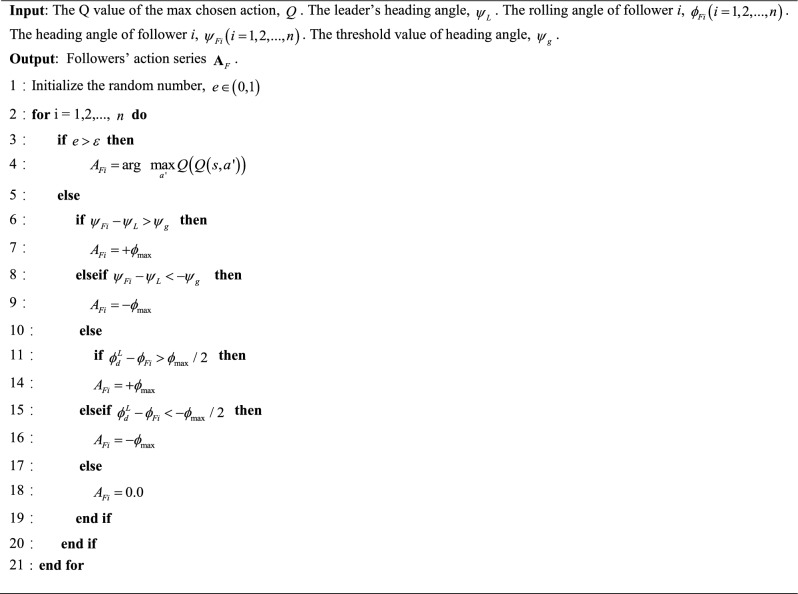
Algorithm 1: $\varepsilon$-imitation action selection strategy.

#### DQN method

**Figure 5:**
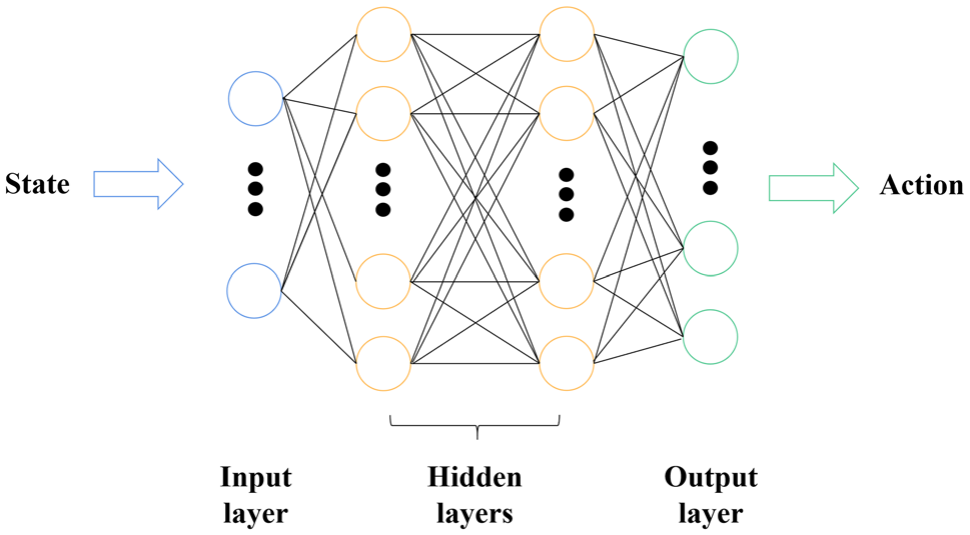
The conceptual structure of a Q-network.

The Q function in the DQN framework is estimated by the utilization of a neural network, known as a Q-network, which is characterized by its weight parameters denoted as $\theta$. In order to assess the Q value, a fully linked Q-network is constructed, as depicted in Fig. 5. At time step $t$, the state of the UAVs is accepted by the input layer of the Q-network. Every individual node within the output layer of the neural network corresponds to the Q value associated with a specific action within the set of all possible actions. The network is comprised of two hidden layers, single input layer and single output layer. Dimensions of the hidden layer are set to 40 × 40, and the training function is specified as Variable Learning Rate Gradient Descent.

UAVs coordination control is accomplished by using the DQN algorithm. Figure 4 illustrates the training process, depicting the key implementation elements of the collaborative control method based on DQN in Algorithm 2.

**Figure:**
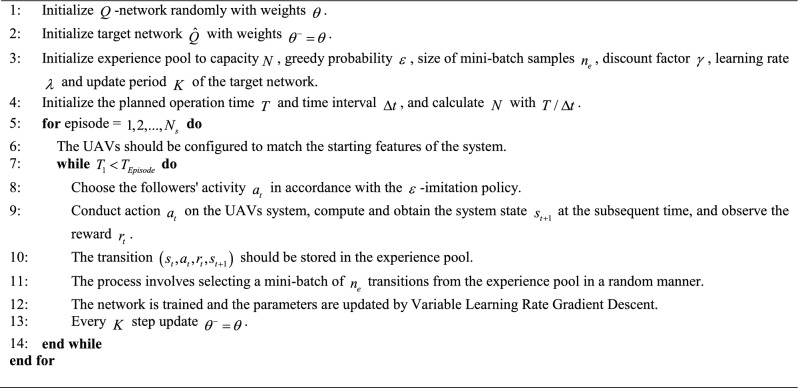
Algorithm 2: DQN method.

## Results and discussion

The training process is finished in MATLAB which includes 50,000 episodes totally. In each episode, the simulation time is 60 s. Before the formal training, a pre-training of 200 episodes is conducted to collect experiential data for batch training. During the training process, the exploration probability $\varepsilon$ linearly decreases from the initial value of 1 to the minimum value of 0.1 over 10,000 episodes, and the update period of the target network $K$ gradually increases from 1000 to 10,000 in the initial 1000 episodes.

The simulation data-set consists of UAVs with a leader and two followers which monitoring the configuration^38^. The parameters pertaining to the dynamics of UAVs are presented here.

Table 1 provides a comprehensive overview of the specific parameter configurations for the DQN algorithm.

Table 2 displays the physical characteristics of both the leader and the follower.

**Table 2:** Arguments of the leader and the follower.

	Leader	Follower 1	Follower 2
Initial position (m)	(0,0)	(30,40)	(− 30,40)
$V_{0}$ (m/s)	10	10	10
$\psi_{{_{0} }}$ (°)	0.0	0.0	0.0
$\phi_{0}$ (°)	0.0	0.0	0.0

In order to assess the efficacy of the DQN-based method utilized in this study, an average reward $R_{Avg}$ was established as the evaluative standard. The variable $R_{Avg}$ is formally delimited as^31^,

14 $$\begin{gathered} N_{E} = \frac{{t_{E} }}{\Delta t} \hfill \\ R_{Avg} = \frac{1}{{N_{Avg} N_{E} }}\sum\limits_{n = 1}^{{N_{Avg} }} {\sum\limits_{t = 1}^{{N_{E} }} {r_{n,t} } } \hfill \\ \end{gathered}$$

where $r$ represents the immediate reward in Eq. (7).

**Figure 6:**
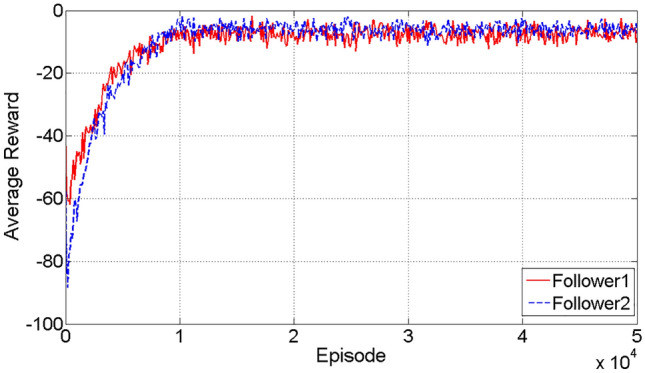
The variation of the average rewards.

Figure 6 illustrates the fluctuations in the average rewards across all training episodes. The results indicate that the average rewards reach a state of convergence following around 10,000 training events for a pair of followers. From what is shown in Fig. 6, after the curves exhibit convergence, the average rewards tend to hover around a value of − 7.

**Figure 7:**
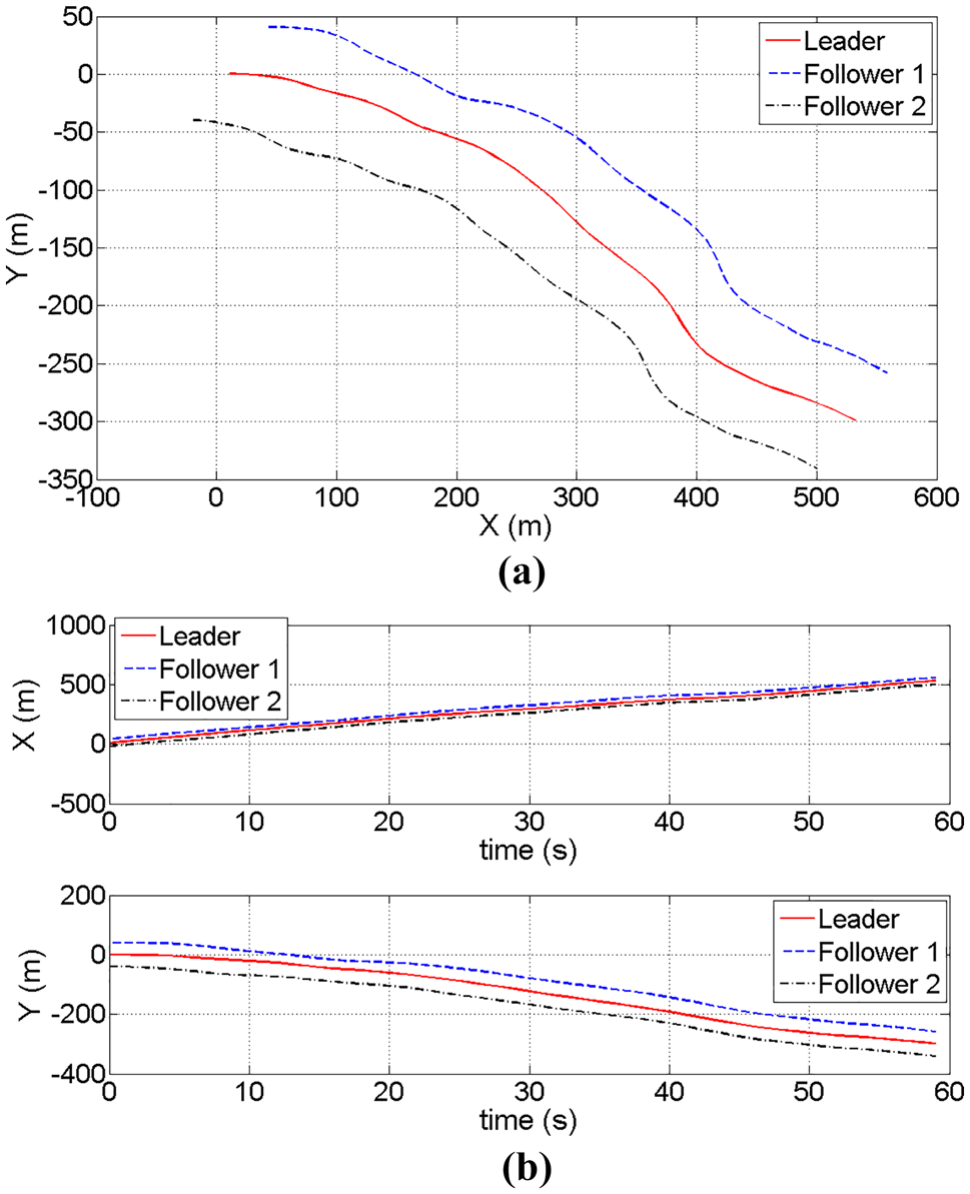
The relative motion relationship of the leader and the followers. (**a**) The trajectory of the leader and the followers. (**b**) The relative distance change in X and Y direction of the leader and the followers.

According to the highest reward of the episode, the results are shown as follows. Figure 7 shows the trajectory curves and distance change in different directions of leader and a pair of followers. Based on the observed patterns of the curves, it can be inferred that the followers are consistently converging towards the trajectory of the leader in both the X and Y directions.

**Figure 8:**
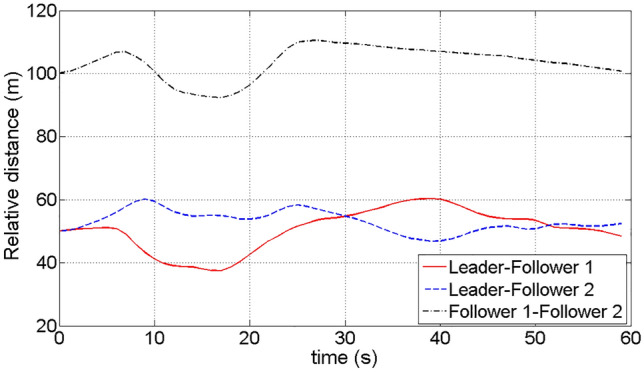
The relative distance variation of the leader and the followers.

Figure 8 depicts the comparative distance-time profiles of the leader and the followers. The adjustment of the reward function reveals the establishment of a secure distance range of 40–60 m between the leader and the followers. Moreover, the followers also maintained a safe relative distance from each other, and there is no risk of collision during the simulation.

**Figure 9:**
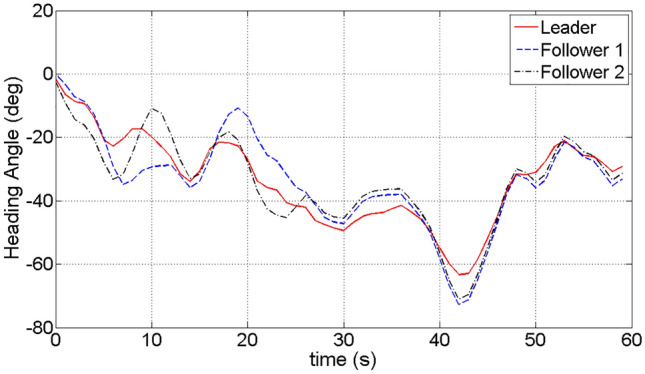
The heading angle change between the leader and the followers.

In Fig. 9, it shows that the heading angle of followers changes with the leader. Along with the heading angle change of the leader, followers gradually adjust their heading angle and fly with the leader in a similar trends. After 45 s of simulation time, it shows that the heading angle changes of the leader and the followers tend to be consistent.

**Figure 10:**
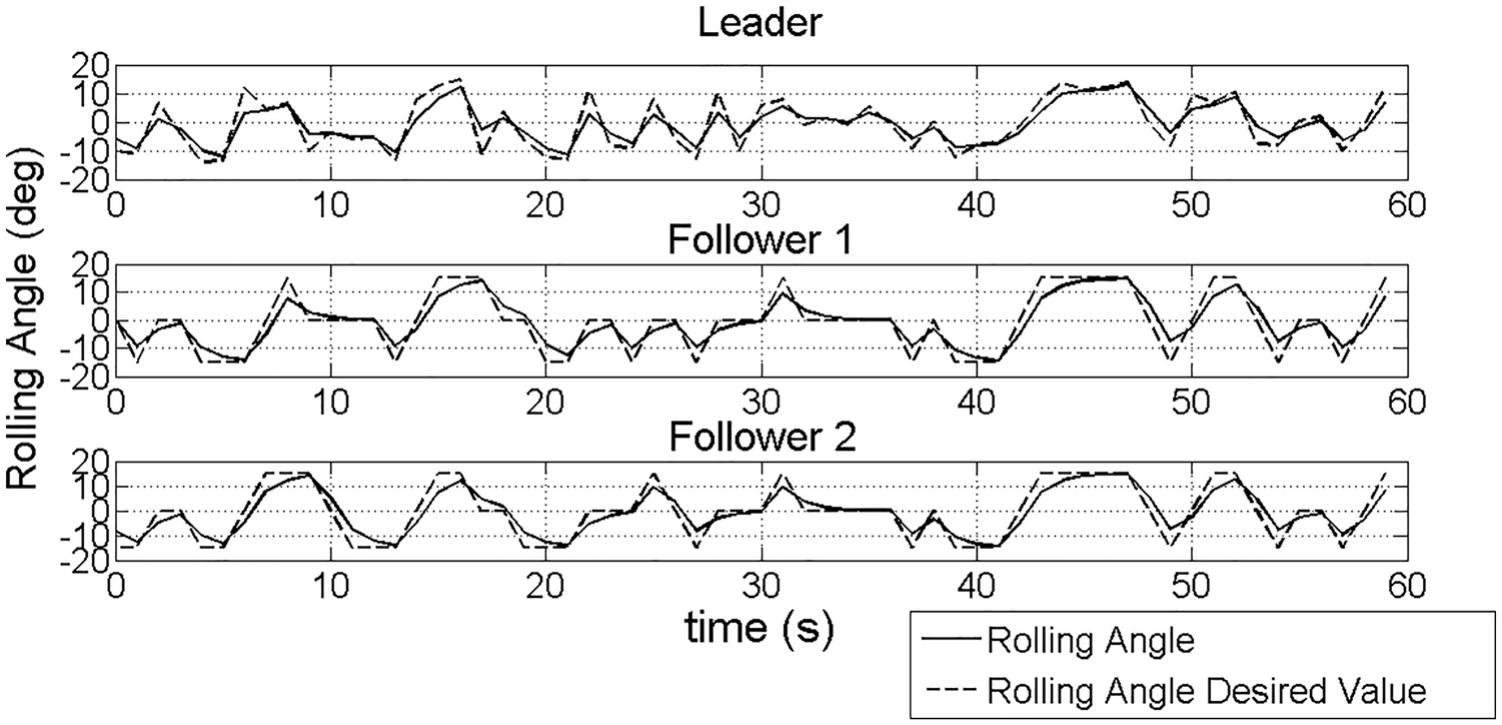
The comparison of rolling angle and its desired value for the leader and the followers.

Figure 10 illustrates the changes in rolling angle and its desired value of followers. In order to collaborate with the leader to fly within a safe distance, the rolling angle controls of followers are adjusted continuously according to the action space and $\varepsilon$-imitation action selection strategy. As is shown in this figure, followers’ rolling angle and commands are all changing with the leader's roll angle which are in a range from − 20° to 20°.

## Conclusion

The objective of this study was to devise an innovative approach for addressing the challenges posed by nondeterminacy, non-linearity, systematical error, and disturbances in the modeling of UAVs. A novel intelligent control strategy was proposed for the cooperative control job of UAVs, utilizing a DQN algorithm. The proposed system joint states encompassed the relative position, heading angle and rolling angle in the formation of UAVs, as defined in the environmental context. Subsequently, a MDP model was modified to depict the collaborative control process of UAVs, using the fundamental scheme of RL. Afterwards, the comprehensive DQN algorithm was presented, encompassing the fundamental structure, the -imitation action selection technique, and a complete account of the algorithm. In order to assess the effectiveness and practicality of the DQN control technique mentioned in this research, a simulation experiment was conducted.

Based on the outcomes of the simulation, it can be observed that the algorithm based on DQN exhibits a distinctive level of behavior in the context of cooperative control of UAVs. The average total reward profile demonstrates a satisfactory level of astringency, indicating that the collaborative controller design meets the necessary requirements for the relative kinematic link among different nodes in the formation. In subsequent research endeavors, there will be an expansion towards the development of a high-fidelity hardware-in-the-loop simulation system. This system will be designed to assess the efficacy and adaptability of the DQN-based method. The control algorithm that has been trained within the numerical emulation environment can be seamlessly shifted to the hardware-in-the-loop system with minimal argument adjustments required.

## Data Availability

The code alongside the datasets used and generated during the current study are available from the corresponding author on reasonable request.

## References

[1] Zhou, X. *et al.* Swarm of micro flying robots in the wild. *Sci. Robot.* **7**, eabm5954. 10.1126/scirobotics.abm5954 (2022). 35507682 10.1126/scirobotics.abm5954

[2] Azar, A. T. *et al.* Drone deep reinforcement learning: A review. *Electronics.* **10**, 999. 10.3390/electronics10090999 (2021). 10.3390/electronics10090999

[3] Oroojlooy, A. & Hajinezhad, D. A review of cooperative multi-agent deep reinforcement learning. *Appl. Intell.* **53**, 13677–13722. 10.1007/s10489-022-04105-y (2023). 10.1007/s10489-022-04105-y

[4] Xu, D. & Chen, G. Autonomous and cooperative control of UAV cluster with multi-agent reinforcement learning. *Aeronaut. J.* **126**, 932–951. 10.1017/aer.2021.112 (2022). 10.1017/aer.2021.112

[5] Eslamiat, H., Li, Y., Wang, N., Sanyal, A. K., & Qiu, Q. Autonomous waypoint planning, optimal trajectory generation and nonlinear tracking control for multi-rotor UAVs. In *18th European Control Conference (ECC)* 25–28. 10.23919/ECC.2019.8795855 (2019).

[6] Hu, J. *et al.* Autonomous maneuver decision making of dual-UAV cooperative air combat based on deep reinforcement learning. *Electronics.* **11**, 467. 10.3390/electronics11030467 (2022). 10.3390/electronics11030467

[7] Wang, Y., Ren, T., & Fan, Z. Autonomous Maneuver Decision of UAV Based on Deep Reinforcement Learning: Comparison of DQN and DDPG. In *34th Chinese Control and Decision Conference (CCDC)* 4857–4860. 10.1109/ccdc55256.2022.10033863 (2022).

[8] Yang, X., Gao, H., Wang, C., *et al.* Formation change strategy of multiple UAVs based on improved DQN. In *International Conference on Guidance, Navigation and Control* 4632–4642. 10.1007/978-981-19-6613-2_449 (2022).

[9] Rodriguez-Ramos, A. *et al.* A deep reinforcement learning strategy for UAV autonomous landing on a moving platform. *J. Intell Robot Syst.* **93**, 351–366. 10.1007/s10846-018-0891-8 (2019). 10.1007/s10846-018-0891-8

[10] Akhloufi, M. A., Arola, S. & Bonnet, A. Drones chasing drones: Reinforcement learning and deep search area proposal. *Drones.* **3**, 58. 10.3390/drones3030058 (2019). 10.3390/drones3030058

[11] Singh, G., Lofaro, D. M., & Sofge, D. Pursuit-evasion with Decentralized Robotic Swarm in Continuous State Space and Action Space via Deep Reinforcement Learning. In *12th International Conference on Agents and Artificial Intelligence* 226–233. 10.5220/0008971502260233 (2020).

[12] Xu, D. *et al.* Morphing control of a new bionic morphing UAV with deep reinforcement learning. *Aerosp. Sci. Technol.* **92**, 232–243. 10.1016/j.ast.2019.05.058 (2019). 10.1016/j.ast.2019.05.058

[13] Wada, D., Araujo-Estrada, S. A. & Windsor, S. Unmanned aerial vehicle pitch control under delay using deep reinforcement learning with continuous action in wind tunnel test. *Aerospace.* **8**, 258. 10.3390/aerospace8090258 (2021). 10.3390/aerospace8090258

[14] Zhou, S., Li, B., Ding, C., *et al.* An efficient deep reinforcement learning framework for uavs. In *21st International Symposium on Quality Electronic Design* 323–328. 10.1109/ISQED48828.2020.9136980 (2020).

[15] Zhao, Y. *et al.* Reinforcement learning-based collision avoidance guidance algorithm for fixed-wing uavs. *Complexity.* **2021**, 1–12. 10.1155/2021/8818013 (2021). 10.1155/2021/8818013

[16] Moon, J. *et al.* Deep reinforcement learning multi-UAV trajectory control for target tracking. *IEEE Internet Things J.* **8**, 15441–15455. 10.1109/JIOT.2021.3073973 (2021). 10.1109/JIOT.2021.3073973

[17] Liu, C. H. *et al.* Energy-efficient UAV control for effective and fair communication coverage: A deep reinforcement learning approach. *IEEE J. Sel. Area Comm.* **36**, 2059–2070. 10.1109/JSAC.2018.2864373 (2018). 10.1109/JSAC.2018.2864373

[18] Wang, L. *et al.* Multi-agent deep reinforcement learning-based trajectory planning for multi-UAV assisted mobile edge computing. *IEEE Trans. Cogn. Commun. Netw.* **7**, 73–84. 10.1109/TCCN.2020.3027695 (2020). 10.1109/TCCN.2020.3027695

[19] Zhang, Y., Zhang, Y., & Yu, Z. Path following control for UAV using deep reinforcement learning approach. *Guid. Navig. Control.* **1**, 2150005. 10.1142/S2737480721500059 (2021).

[20] Wang, Y. *et al.* Cooperative USV–UAV marine search and rescue with visual navigation and reinforcement learning-based control. *ISA Trans.* **137**, 222–235. 10.1016/j.isatra.2023.01.007 (2023). 36801140 10.1016/j.isatra.2023.01.007

[21] Wan, K. *et al.* Robust motion control for UAV in dynamic uncertain environments using deep reinforcement learning. *Remote Sens.* **12**, 640. 10.3390/rs12040640 (2020). 10.3390/rs12040640

[22] Xu, D., & Chen, G. Reinforcement learning for autonomous morphing control and cooperative operations of UAV cluster. In *Deep Learning for Unmanned Systems* (ed 1st) 309–354 (Switzerland, 2021). 10.1007/978-3-030-77939-9_9.

[23] Tožička, J., Szulyovszky, B., & de Chambrier, G., *et al.* Application of deep reinforcement learning to UAV fleet control. In *2018 Intelligent Systems Conference* 1169–1177. 10.1007/978-3-030-01057-7_85 (2018).

[24] Wang, R. *et al.* Least global position information based control of fixed-wing UAVs formation flight: Flight tests and experimental validation. *Aerosp. Sci. Technol.* **140**, 108473. 10.1016/j.ast.2023.108473 (2023). 10.1016/j.ast.2023.108473

[25] Dinelli, C. *et al.* Configurations and applications of multi-agent hybrid drone/unmanned ground vehicle for underground environments: A review. *Drones.* **7**, 136. 10.3390/drones7020136 (2023). 10.3390/drones7020136

[26] Cai, W. *et al.* Cooperative artificial intelligence for underwater robotic swarm. *Robot. Auton. Syst.* **164**, 104410. 10.1016/j.robot.2023.104410 (2023). 10.1016/j.robot.2023.104410

[27] Xu, W. & Cao, N. Research on the strategies for collision avoidance of multi-UAV with three dimensional formation in combination of consensus algorithm and uniform flow. *IET Control Theory Appl.* **00**, 1–23. 10.1049/cth2.12521 (2023). 10.1049/cth2.12521

[28] Shen, J. *et al.* Typical fault estimation and dynamic analysis of a leader-follower unmanned aerial vehicle formation. *Int. J. Aerosp. Eng.* **2021**, 1–16. 10.1155/2021/6656422 (2021). 10.1155/2021/6656422

[29] Wang, B. L., Li, S. G., Gao, X. Z. & Xie, T. UAV swarm confrontation using hierarchical multi-agent reinforcement learning. *Int. J. Aerosp. Eng.* **2021**, 1–12. 10.1155/2021/3360116 (2021). 10.1155/2021/3360116

[30] Luo, L. *et al.* Grpavoid: Multigroup collision-avoidance control and optimization for UAV swarm. *IEEE Trans. Cybern.* **53**, 1776–1789. 10.1109/TCYB.2021.3132044 (2023). 34936562 10.1109/TCYB.2021.3132044

[31] Xiang, X. J. *et al.* Towards coordination control for fixed-wing UAV formation through deep reinforcement learning. *Acta Aeronaut. Astronaut. Sin.* **42**(4), 524009–524009. 10.7527/S1000-6893.2020.24009 (2021). 10.7527/S1000-6893.2020.24009

[32] Wei, Z. C. *et al.* A Q-learning algorithm for task scheduling based on improved SVM in wireless sensor networks. *Comput. Netw.* **161**, 138–149. 10.1016/j.comnet.2019.06.006 (2019). 10.1016/j.comnet.2019.06.006

[33] Wen, X. Q. & Xu, Z. A. Wind turbine fault diagnosis based on Relief FPCA and DNN. *Expert. Syst. Appl.* **178**, 115016. 10.1016/j.eswa.2021.115016 (2021). 10.1016/j.eswa.2021.115016

[34] Liu, W., Su, S., Tang, T. & Wang, X. A DQN-based intelligent control method for heavy haul trains on long steep downhill section. *Transp. Res. Part C Emerg. Technol.* **129**, 103249. 10.1016/j.trc.2021.103249 (2021). 10.1016/j.trc.2021.103249

[35] Mnih, V. *et al.* Human-level control through deep reinforcement learning. *Nature.* **518**, 529–533. 10.1038/nature14236 (2015). 25719670 10.1038/nature14236

[36] Huang, H. J. *et al.* Deep reinforcement learning for UAV navigation through massive MIMO technique. *IEEE Trans. Veh. Technol.* **69**, 1117–1121. 10.1109/TVT.2019.2952549 (2020). 10.1109/TVT.2019.2952549

[37] Shen, J., Zhang, B. K., Zhu, Q. Y. & Chen, P. Y. A deep-network-based collaborative control research for smart ammunition formation. *Int. J. Aerosp. Eng.* **2022**, 1–15. 10.1155/2022/2021693 (2022). 10.1155/2022/2021693

[38] Shen, J., Zhu, Q. Y., Xu, L., Chen, G. G., Tian, X. L., & Yan, X. L. Research on dynamic simulation and collaborative control of smart ammunition formation. In *IEEE International Conference on Mechatronics and Automation (ICMA)* 1488–1492. 10.1109/ICMA49215.2020.9233661 (2020).

